# Association Usefulness

**Published:** 1900-10

**Authors:** 


### EDITORIALS.

### ASSOCIATION USEFULNESS.

THE wealth of a scientific body is only to be measured by the scope of its work.

Its monetary gatherings are ever to be expended in broadening the sphere of its usefulness in the pressing questions of the hour.

Sanitary medicine, bacteriological research, laboratory investigations, national standing, State medicine, municipal problems from a health point of view have been fruitful topics of consideration in American veterinary medicine for many years. Many victories have been achieved, progress has been made, questions of much moment have been solved; but the growth in numbers and strength add greater duties, more serious responsibilities to those charged with official direction than ever before; and how an organized army of nearly five hundred men are to best serve their profession in associated interest during the coming year will be a serious problem for those in official capacity to determine. Surely the stewardship of so large and vigorous a body is an important one. The honor of its chief directorate is freighted with greater and greater responsibilities in a profession historical to-day in its phenomenal growth and influence throughout our land.

